# Thoracic Ultrasound for Pre-Procedural Dynamic Assessment of Non-Expandable Lung: A Non-Invasive, Real-Time and Multifaceted Diagnostic Tool

**DOI:** 10.3390/jcm14062062

**Published:** 2025-03-18

**Authors:** Guido Marchi, Federico Cucchiara, Alessio Gregori, Giulia Biondi, Giacomo Guglielmi, Massimiliano Serradori, Marco Gherardi, Luciano Gabbrielli, Francesco Pistelli, Laura Carrozzi

**Affiliations:** 1 Pulmonology Unit, Cardiothoracic and Vascular Department, University Hospital of Pisa, Via Paradisa 2, 56124 Pisa, Italy; 2 Department of Surgical, Medical and Molecular Pathology and Critical Care Medicine, University of Pisa, 56126 Pisa, Italy

**Keywords:** thoracic ultrasound (TUS), non-expandable lung (NEL), lung entrapment, trapped lung, pleural manometry, malignant pleural effusion (MPE), indwelling pleural catheter (IPC), thoracentesis, diagnosis, pleural effusion (PE)

## Abstract

Non-expandable lung (NEL) occurs when the lung fails to fully re-expand after pleural fluid drainage, complicating management and limiting therapeutic options. Diagnosis, based on clinical symptoms, pleural manometry, and traditional imaging, is often delayed to the peri- or post-procedural stages, leading to improper management, complications, and higher healthcare costs. Therefore, early, pre-procedural diagnostic methods are needed. Thoracic ultrasound (TUS) has emerged as a non-invasive tool with the potential to enhance diagnostic accuracy and guide clinical decisions, yet, it remains inadequately studied within the context of NEL. We conducted a non-systematic narrative review using a structured methodology, including a comprehensive database search, predefined inclusion criteria, and QUADAS-2 quality assessment. This approach ensured a rigorous synthesis of evidence on TUS in NEL, with the aim of identifying knowledge gaps and guiding future studies. Non-invasive, real-time, bedside M-mode TUS has demonstrated efficacy in predicting NEL prior to thoracentesis by detecting an absent sinusoidal sign and reduced atelectatic lung movement. Emerging experimental techniques, including 2D shear wave elastography (SWE), speckle tracking imaging (STI) strain analysis, the lung/liver echogenicity (LLE) ratio, TUS assessment of dynamic air bronchograms, and pleural thickening evaluation, show additional potential to enhance pre-procedural NEL detection. However, all these methods have significant limitations that require further comprehensive investigation. Despite their significant promise, TUS modalities for early NEL detection still require rigorous validation and standardization before broad clinical use. A multimodal diagnostic approach, combining clinical manifestations, pleural manometry, radiologic and ultrasonographic findings, along with emerging techniques (once fully validated), may provide the most extensive framework for NEL. Regardless of advancements, patient-centered care and shared decision-making remain essential. Further research is needed to improve outcomes, reduce healthcare costs, and enhance long-term treatment strategies.

## 1. Introduction

Non-Expandable Lung (NEL) is an umbrella term encompassing a spectrum of pathological conditions in which the lung fails to expand fully against the chest wall, preventing the physiological apposition of the visceral and parietal pleura. This phenomenon significantly affects pulmonary function and clinical management, particularly in patients with pleural effusion (PE) and thoracic malignancies. The primary causes of NEL include: (1) endobronchial obstruction leading to lobar collapse or chronic lung atelectasis, (2) reduced pulmonary compliance due to extensive pulmonary fibrosis and scarring, and (3) visceral pleural constriction because of pleural disease [1,2].

From a pathophysiological standpoint, NEL can be classified into two distinct entities: trapped lung and lung entrapment [2,3]. Trapped lung results from the formation of a fibrotic pleural peel in the absence of ongoing pleural disease, which mechanically restricts lung expansion. As a consequence, the pleural space is unable to achieve normal negative pressure after thoracentesis. In contrast, lung entrapment occurs in the setting of active pleural pathology, such as malignancy or infection, which leads to an inflammatory process that progressively limits pulmonary re-expansion. These mechanisms underline the significant impact of NEL on clinical outcomes, including reduced symptom relief following pleural drainage, increased risk of procedural complications, and suboptimal response to pleurodesis [3].

The diagnosis of NEL remains a challenge in clinical practice. Early detection, particularly pre-procedural identification, would enable optimization of the therapeutic approach (e.g., early placement of an indwelling pleural catheter (IPC) or avoidance of repeated pleural aspirations), potentially improving the quality of life for patients affected by this condition [4]. In contrast, a peri- or post-procedural diagnosis may expose patients to worsening respiratory symptoms, a decline in overall quality of life, and complications that may require hospitalization [5].

This article critically evaluates the current standards for diagnosing NEL and explores emerging diagnostic techniques, with a particular focus on pre-procedural assessment, aiming to identify existing knowledge gaps and provide direction for future research.

Conventional imaging modalities, such as chest radiography (CXR) and computed tomography (CT), provide structural information for post-procedural evaluation but are limited in their ability to differentiate the various etiologies of lung non-expandability [2,6]. CXR may miss subtle changes, and its two-dimensional view can obscure pleural details due to overlapping structures. Both CXR and CT lack dynamic evaluation (e.g., absence of lung sliding), potentially underestimating pleural adhesions’ complexity. Pleural manometry is useful in distinguishing trapped lung from lung entrapment but is underutilized due to operator dependence, specialized equipment, and extended procedure time [7,8,9,10]. These limitations hinder its widespread use, especially in resource-limited settings, highlighting the need for further research and standardization.

In recent years, thoracic ultrasound (TUS) has gained prominence as a non-invasive, real-time imaging modality for detecting dynamic pleural and pulmonary abnormalities indicative of NEL [10,11,12]. TUS provides a dynamic, bedside assessment, overcoming some of the limitations of conventional imaging and offering more detailed insights into pleural mechanics.

Among the various sonographic techniques, M-mode ultrasound has emerged as a valuable tool for predicting lung re-expansion prior to intervention, refining pre-procedural assessment, and guiding therapeutic decisions [13,14,15,16,17,18,19,20,21,22,23].

Despite these above-mentioned advancements, no universally accepted gold standard for NEL pre-procedural diagnosis has been established.

Emerging experimental methods have shown potential in enhancing diagnostic accuracy [15,21,23,24,25]. Speckle tracking imaging (STI) strain analysis evaluates pleural dynamics by measuring tissue deformation. The lung/liver echogenicity (LLE) Ratio compares echogenicity between the lung and liver to predict lung re-expansion after pleural drainage. Two-dimensional shear wave elastography (SWE) assesses tissue stiffness. TUS-based evaluations, including dynamic air bronchograms and pleural thickening, offer valuable insights into lung function and structure [15,21,23,24,25]. However, the clinical utility of these methods is still under investigation, and further validation through larger studies is needed to fully establish their relevance and accuracy.

Given the significant clinical and prognostic implications of NEL, there is a critical need for research on early, pre-procedural, non-invasive diagnosis. This review aims to provide an updated synthesis of the current literature on NEL, focusing on different diagnostic modalities, including conventional TUS and emerging experimental techniques.

Additionally, we will explore existing controversies and divergent hypotheses regarding optimal management strategies and patient-centered care approaches.

A deeper understanding of NEL is crucial for improving diagnostic accuracy, informing appropriate treatment decisions, reducing healthcare costs, minimizing patient complications, and ultimately enhancing patient outcome.

## 2. Methods

A non-systematic narrative literature review was conducted to assess the current evidence on the use of TUS in the pre-procedural assessment of NEL, aiming to identify knowledge gaps and guide future studies. To summarize and synthesize key findings from various studies, PubMed, Google Scholar, Scopus, and Web of Science databases were investigated between 1 January 2025 and 20 February 2025, scouting for relevant research articles published up to December 2024. Key search terms like “thoracic ultrasound” OR “TUS”, “non-expandable lung” OR “NEL” OR “lung entrapment” OR “trapped lung”, “pleural effusion”, “malignant pleural effusion” OR “MPE”, as well as “chest X-ray” OR “CXR”, “chest CT”, “pleural manometry”, “indwelling pleural catheter” OR “IPC”, “thoracentesis” and “pneumothorax” OR “PNX”, were combined using appropriate Boolean operators. Search strings were customized to each database’s syntax.

Additional relevant articles were identified through citation tracking of key papers and review of reference lists from included studies.

This review includes research articles published in English, reporting clinical findings about the use of thoracic ultrasound (TUS), pleural manometry, and radiological techniques in the pre-, peri-, and post-procedural assessment of non-expandable lung (NEL). No geographical restrictions were applied: studies from Asia, Africa, Europe, North and South America, and Oceania were all considered. Studies were included in the review if they met the following criteria: (1) investigation of TUS applications in the adult human populations with pleural effusion; (2) examination of TUS findings for predicting and managing NEL; (3) evaluation of chest radiography (CXR), computed tomography (CT), and/or pleural manometry in adult human population with NEL; (4) comparison of TUS diagnostic performance against CXR, CT, and/or pleural manometry; and (5) reporting of emerging diagnostic techniques and their performance metrics in investigating and managing NEL in the adult human population. Both peer-reviewed journal publications and data from abstracts presented at international scientific meetings (even if not yet published in full), were considered eligible. Additionally, relevant case reports, reviews, and editorials identified through citation tracking or reference lists of selected key papers were included to address concepts pertinent to the review. Studies were excluded if they were not in the English language, limited to animal or in vitro experiments, did not address NEL assessment or management, lacked clear methodology, contained insufficient technical details, or were non-peer-reviewed (except for relevant conference abstracts from recognized international meetings).

Article selection followed a two-step process. First, five independent reviewers (G.M., F.C., A.G., G.B., and G.G.) conducted a preliminary abstract screening. Subsequently, a full-text review was performed for all relevant studies deemed potentially eligible by each screener. When discrepancies in article selections occurred, they were resolved through consensus discussion also involving experienced clinicians (M.S., M.G., L.G., F.P., and L.C.). Studies unanimously approved were ultimately selected.

Given the methodological and clinical heterogeneity of the included studies, data extraction was structured to capture essential study characteristics (e.g., study design, selected population, diagnostic modality, and related performance metrics), allowing for a qualitative synthesis rather than a meta-analysis. Findings were organized based on TUS modality and diagnostic accuracy parameters to facilitate meaningful interpretation.

Also, to ensure methodological rigor, since the search aimed to identify a comprehensive range of studies addressing these topics, enhancing the understanding of TUS potential applications in the assessment of NEL conditions and stimulating further valid research in this area. A Quality Assessment of Diagnostic Accuracy Studies-2 (QUADAS-2) framework [26] was applied whenever possible to evaluate the risk of bias and reliability of included diagnostic studies. The framework enabled the assessment across four critical domains: (1) Patient Selection, including recruitment methods and inclusion/exclusion criteria; (2) Index Test, evaluating whether TUS methodology was properly described and assessed independently of reference standard results; (3) Reference Standard, determining whether studies compared TUS results with pleural manometry or other established standards such as CXR and CT imaging; as well as (4) Flow & Timing, reckoning whether all participants were properly accounted for, including any lost to follow-up, and whether tests were conducted within appropriate time frames.

Studies were rigorously evaluated and rated as having low, high, or unclear risk of bias. Those demonstrating consistent methodological robustness were prioritized, while those with limitations, such as inadequate blinding or inconsistencies in reference standards, were included but interpreted with appropriate caution.

This balanced approach ensured a comprehensive yet critical appraisal of the evidence, maintaining transparency and reproducibility in accordance with the SANRA guidelines for narrative reviews [27].

## 3. Non-Expandable Lung: Clinical Significance of an Understudied Topic

The clinical significance of NEL in the context of malignant pleural effusion (MPE) is paramount, as it directly impacts the management and prognosis of affected patients. NEL, which can arise from trapped lung or lung entrapment due to various pathological processes like malignancy or infection, complicates the effective re-expansion of the lung and often leads to suboptimal response to standard treatments like pleurodesis [1]. This condition can cause persistent symptoms such as breathlessness and chest pain, severely affecting a patient’s quality of life (QoL).

Despite its clear relevance, the clinical understanding, diagnosis, and management of NEL remain largely underexplored to this day. While much of the research on MPE focuses on improving overall survival and treatment efficacy, the specific mechanisms of NEL and its impact on clinical outcomes are still inadequately studied [28]. This underrepresentation in research highlights a crucial gap in our knowledge, which, if addressed, could lead to more targeted and effective management strategies.

Advancements in diagnostic techniques, such as TUS, pleural manometry, and emerging imaging methods, provide promising opportunities to better identify and assess NEL. However, the lack of consensus on a diagnostic gold standard means that many patients may still be misdiagnosed or improperly treated, further underscoring the need for comprehensive, high-quality research.

## 4. Pre-, Peri-, and Post-Procedural Non-Expandable Lung Assessment

### 4.1. Clinical Symptoms

Patients presenting with NEL frequently report progressive dyspnea arising from the chronic accumulation of pleural fluid, which gradually reduces the effective lung volume [1,29,30,31,32,33]. In addition, a non-productive cough is reported as a nonspecific indicator of ongoing pleural irritation [1,29,30,31,32,33], and pleuritic chest pain also commonly stems from both the underlying inflammatory milieu (secondary to malignancy, infection, or post-inflammatory fibrosis) and the mechanical strain imposed on the pleural surfaces [1,29,30,31,32,33]. On physical examination, decreased or absent breath sounds over the affected hemithorax are commonly noted [1,29,30,31,32,33].

Symptom relief is generally expected in patients undergoing thoracentesis, but not in those with NEL. Patients with NEL may instead notice no benefit or even experience a sudden onset of chest discomfort immediately following fluid withdrawal [2,8,29,34]. This paradoxical response suggests the unyielding nature of the entrapped lung. Not surprisingly, indeed, the pathophysiology of NEL involves the formation of a fibrous peel on the visceral pleura due to chronic inflammation, which ultimately prevents lung re-expansion even after effective fluid drainage [2].

Consequently, the post-procedural persistence of dyspnea and unchanged auscultatory findings definitively reinforce the diagnosis of NEL [2,8,29,34]. This diagnosis can be further supported by peri-procedural monitoring tools, which provide real-time findings to enhance diagnostic accuracy and guide subsequent management strategies.

### 4.2. Pleural Manometry

**Figure 1:**
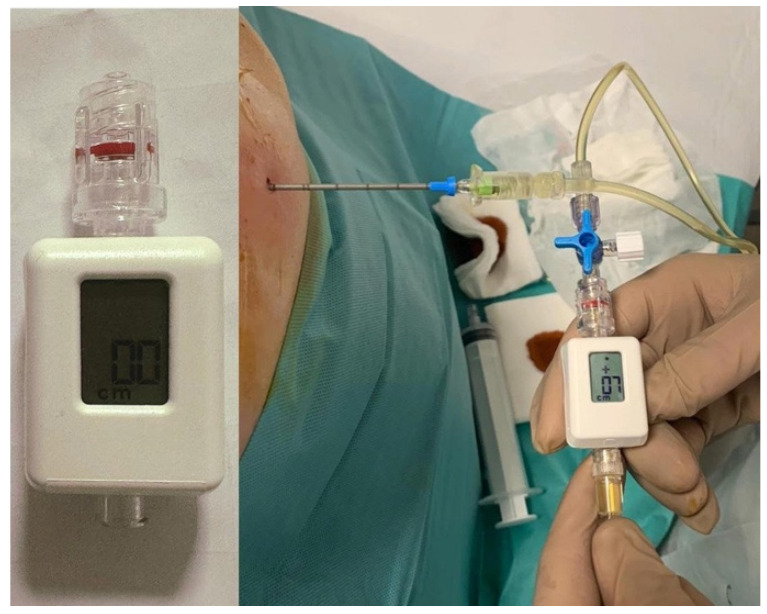
Digital pleural manometer. During thoracentesis, a pressure transducer connected to the drainage catheter measures changes in intrapleural pressure.

Pleural manometry has emerged as an invaluable adjunct in the peri-procedural assessment of NEL [7,8,9]. During thoracentesis, a pressure transducer attached to the drainage catheter measures intrapleural pressure changes, thereby generating a pressure-volume curve during fluid removal (Figure 1) [9,35]. The numerical relationship between the volume of fluid removed and the respective decline in pleural pressure is referred to as pleural elastance (PEL) and is calculated as a derivative of pleural pressure drop versus the derivative of the volume of pleural fluid removed (dP/dV) [9,35]. Based on 247 pleural manometry procedures reported by Huggins et al. [25] and 192 procedures by Heidecker et al. [36], normal PEL is defined as ranging from 0.5 to 14.5 cmH_2_O/L (mean ± 2 SD), with values exceeding 14.5 cmH_2_O/L considered elevated [9,35].

Under normal circumstances, as pleural fluid is withdrawn, pleural pressure decreases gradually. In contrast, in patients with NEL, the pressure-volume curve may either exhibit a steep decline (characteristic of a trapped lung) or reach a plateau (as seen in lung entrapment where partial re-expansion occurs during the initial phase of fluid removal [9,35]. These aberrant patterns reflect the lung’s inability to expand due to the presence of a fibrous peel [2,9,35].

The diagnostic weight of the technique lies right in its ability to provide real-time information on lung mechanics, aiming to predict the likelihood of lung re-expansion and guide appropriate intervention strategies. As an example, by measuring PEL in patients with MPE, Martin et al. found measures > 14.5 cmH_2_O/L to be 100% sensitivity and 67% specificity in identifying NEL, allowing addressing of patients to IPC insertion instead of pleurodesis [37]. Similarly, Masoud et al. further investigated PEL predictive value in pleurodesis; they found that, among 40 patients, those with successful treatment had significantly lower PEL (8.38 ± 2.65 vs. 18.29 ± 4.65 cmH_2_O/L), while ROC analysis showing PEL > 14.5 cmH_2_O/L as a strong predictor of pleurodesis failure (having 94% sensitivity, 100% specificity, and 0.94 area under the curve) [38].

Pleural manometry also serves a critical role in preventing complications associated with large-volume thoracentesis (>1.0–1.5 L [9,35]), such as re-expansion pulmonary edema and (hydro-)pneumothorax ex vacuo [39,40]. By monitoring pleural pressure changes during fluid withdrawal, clinicians can determine optimal fluid removal limits and avoid excessively negative pleural pressures leading to complications.

Despite all these merits, pleural manometry faces practical limitations too. The technique is operator-dependent, requires specialized equipment, and can extend medical procedures [9,35]. These constraints may restrict its widespread clinical implementation, particularly in resource-limited settings, suggesting a need for further research and standardization.

### 4.3. Radiologic Findings

While pleural manometry serves as an elegant technique for estimating PEL, radiological evaluation remains fundamental for assessing lung expandability, and radiological features—such as visceral pleural thickening, septated pleural effusion, ipsilateral volume loss, lobar atelectasis, basal pneumothorax, as well as pneumothorax ex vacuo, or the inability to achieve complete lung re-expansion after fluid withdrawal—provide valuable diagnostic clues [6].

In this regard, both CXR and CT play key roles. Pre-procedural upright and decubitus CXRs as well as CT scans, may already reveal pleural thickening and loculations before fluid removal [2,6].

**Figure 2:**
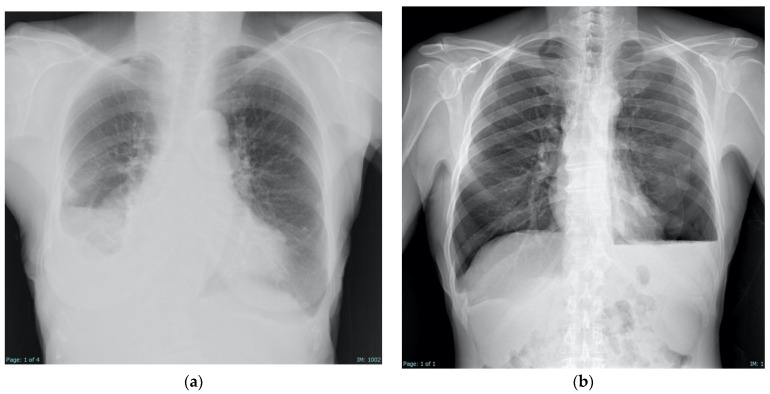
(**a**) Posteroanterior chest radiograph demonstrating hydropneumothorax in a patient with chronic multifactorial pleural effusion and non-expandable lung (NEL). (**b**) Posteroanterior chest radiograph showing hydropneumothorax following pleural drainage in a patient with malignant pleural mesothelioma.

On CXR, pleural fluid may exhibit limited mobility with postural changes, and the paradoxical reduction in the size of the effused hemithorax compared to the contralateral side suggests a locally higher negative pleural pressure, further supporting the diagnosis of a trapped lung [2]. NEL may also be identified radiographically as a (hydro-)pneumothorax ex vacuo, which can be seen on CXR as a basilar pneumothorax (i.e., air in the pleural space that mirrors size and shape of the prior effusion), and often associated to pleural thickening (Figure 2) [41,42].

**Figure 3:**
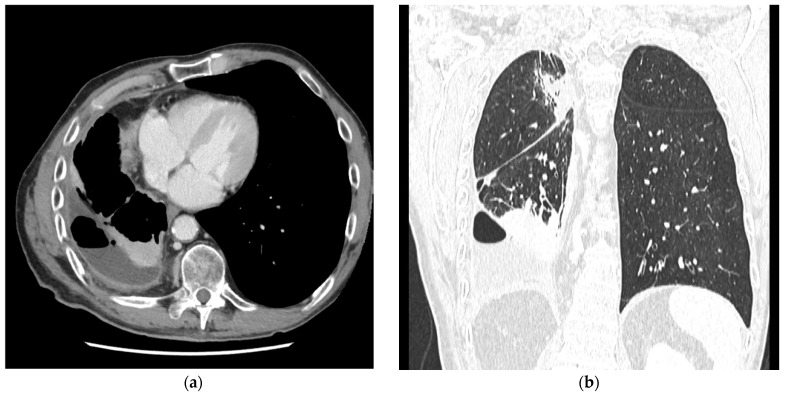
(**a**,**b**) Computed tomography sections demonstrating posterolateral hydropneumothorax due to non-expandable lung in a patient with pulmonary adenocarcinoma and pleural involvement.

Despite its advantages, CXR has significant limitations. Its two-dimensional nature results in overlapping anatomical structures that can obscure subtle pleural pathology, and it lacks the sensitivity to detect early pleural thickening or subtle loculated effusions [43]. In contrast, CT offers superior anatomical detail, allowing comprehensive visualization of visceral pleural thickening, trapped lung, and loculated effusions (Figure 3) [25]. However, CT, like CXR, does not provide dynamic imaging capabilities—an important limitation when assessing functional parameters such as lung sliding during thoracentesis [11,41].

Limitations of these conventional imaging are exemplified by Chopra et al.’s study of 70 MPE cases. Their analysis of post-thoracentesis radiographic findings showed concordance between PEL and radiographic findings in only 71% of cases, with discordant results in 29%, highlighting the challenges in NEL diagnosis [44].

### 4.4. Multimodal Diagnostic Approach

The primary diagnostic challenge remains the fact that NEL is typically detected only after fluid drainage in many cases. As previously outlined, thoracentesis coupled with pleural manometry can be utilized to identify NEL [45]. Reduced lung compliance in the presence of NEL results in amplified changes in pleural pressure, accompanied by heightened pleural elastance [46]. The superiority of pleural manometry over clinical evaluation for NEL (such as chest discomfort during drainage and post-procedural CXRs) and its utility for mid-procedure identification of NEL remain debated. Studies have not demonstrated a reduction in rates of re-expansion failure or chest pain when pleural manometry is used concurrently with thoracentesis [39,40].

Early identification of NEL prior to definitive intervention could offer significant advantages, as first-line IPC insertion is considered the optimal approach in these cases. Non-invasive, real-time, bedside diagnostic modalities that can predict the likelihood of NEL include M-mode TUS during breath-hold, which can detect an absent sinusoidal sign and diminished movement of the atelectatic lung [15,18,20]. These challenges have led to the increased adoption of TUS as a complementary imaging modality [15]. However, this method also has its limitations, including its operator dependence and limited global anatomical perspective [12].

A multimodal strategy incorporating clinical manifestations, pleural manometry, radiologic, and ultrasonographic findings forms a robust diagnostic framework for NEL. However, a conclusive diagnosis is often made primarily during the peri- and post-procedural stages, leading to potential adverse outcomes such as complications, diagnostic delays, and elevated healthcare costs. Consequently, there is an urgent need for further investigation into the development of new early, pre-procedural, non-invasive diagnostic methods for NEL.

A summary table depicting the main imaging evaluation strategies is shown below (Table 1).

## 5. Thoracic Ultrasound Pre-Procedural Non-Expandable Lung Assessment

### 5.1. M-Mode Sinusoid Sign Assessment

Lichtenstein D.A. [13,14] is a prominent figure in the field of TUS, widely recognized for his pioneering contributions to the understanding of various ultrasound signs, including the sinusoid sign. Lichtenstein and colleagues emphasized the critical importance of ultrasound in emergency and critical care settings, where rapid and accurate diagnosis can significantly impact patient management and outcomes. His work, particularly the development of the BLUE (Bedside Lung Ultrasound in Emergency) protocol, has been instrumental in standardizing the use of ultrasound for diagnosing a range of respiratory conditions.

The sinusoid sign is one of the key ultrasound findings described in this protocol. It refers to the characteristic pattern observed when the lung line moves toward the pleural line during inspiration, a phenomenon resulting from the normal expansion of the lung. This motion generates a sinusoidal appearance in the M-mode image, providing valuable insight into pulmonary conditions. The identification of this sign, along with other key ultrasound features, has significantly advanced the diagnostic capabilities of bedside TUS in critically ill patients.

Over the years, several studies have been developed regarding the use of TUS in the pre-procedural assessment of NEL, with particular emphasis on the M-mode sinusoid sign. We provide a chronological review of these studies, which illustrate the evolution of scientific evidence and methodologies related to this diagnostic technique.

**Figure 4:**
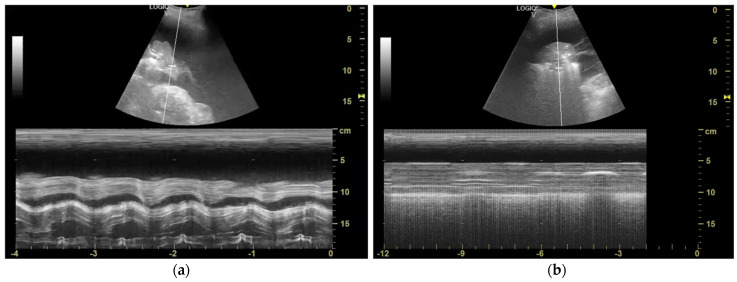
TUS M-mode sinusoid sign assessment. (**a**) Presence of the sinusoid sign in lung atelectasis, indicating respiratory-phase fluctuation in intrapleural distance due to free-flowing effusion around an aerated lung, suggestive of lung expandability. (**b**) Absence of the sinusoid sign, indicating failure of lung de-aeration despite effusion, suggestive of a trapped lung where the atelectatic lung fails to expand and recoil with respiration.

One of the first studies was by Salamonsen et al. [15], who presented a novel approach to diagnosing malignant entrapped lung, a condition that is notoriously difficult to identify prior to PE drainage. This challenge often leads to mismanagement of MPE, particularly when clinicians are unsure whether the lung will re-expand after drainage or remains non-expandable due to entrapment. The research introduced M-mode ultrasound, specifically focusing on the sinusoid sign, as a method to predict the presence of NEL before any invasive procedure is performed (Figure 4). This technique could offer significant advantages in managing MPE, allowing for a more informed choice between interventions such as pleurodesis or the insertion of an IPC.

The study included 81 patients with suspected MPE, all of whom underwent TUS prior to drainage. The ultrasound was conducted using an echocardiogram machine (Vivid E9, GE Healthcare), with cine loops of the atelectatic lower lobe acquired during breath hold at functional residual capacity. This procedure allowed for the analysis of lung motion and deformation through M-mode and speckle tracking imaging (STI). The pleural elastance (PEL) was measured during effusion drainage. The definitive diagnosis of entrapped lung was made based on post-drainage imaging, following a consensus between two interventional pulmonologists, who assessed the radiological results after the procedure.

The results of the study demonstrated that both the total movement and strain of the lung were significantly reduced in cases of entrapped lung. The researchers found that the M-mode ultrasound, particularly through the sinusoid sign, offered a high level of diagnostic reliability. In the development set, the area under the receiver-operating curve (AUC) for diagnosing entrapped lung was 0.86 for STI, 0.79 for M-mode, and 0.69 for PEL. When applying specific cutoffs—6% for STI, 1 mm for M-mode, and 19 cmH_2_O for PEL—the sensitivity and specificity for diagnosing entrapped lung in the validation set were 71%/85%, 50%/85%, and 40%/100%, respectively. These findings underscore the potential of M-mode ultrasound as a reliable tool for predicting entrapped lung before drainage procedures.

The M-mode analysis focused on detecting the sinusoid sign, a characteristic pattern that can identify NEL. When the displacement in M-mode was below 0.8 mm, the study suggested that an IPC would be the most appropriate intervention. Conversely, if the displacement was above 1.2 mm, pleurodesis should be considered as the treatment option. For values between 0.8 and 1.2 mm, the study proposed that a chest tube should be inserted initially, and the decision to proceed with pleurodesis or IPC should be made based on post-drainage radiology, which could confirm the presence or absence of entrapped lung.

Despite the promising results, the study acknowledges several limitations. The diagnosis of isolated lower lobe entrapped lung proved difficult in some cases, and some patients may have been misclassified due to the challenge of distinguishing between slow lung re-expansion and true entrapment. Additionally, in certain instances, pleural fluid was not fully drained, complicating the determination of whether the lung would eventually re-expand. To address these uncertainties, the post-drainage assessments were categorized into “probably entrapped” and “probably free” categories, allowing for additional radiological clues to inform the diagnosis. Furthermore, strain analysis, while a useful technique, is not widely available on portable ultrasound machines at present.

Flora et al. [16] conducted a study to investigate the relationship between the sinusoid sign observed on TUS and pleural manometry during fluid drainage, specifically aiming to assess the utility of the sinusoid sign in predicting NEL physiology. The hypothesis was that the sinusoid sign could serve as a non-invasive and prospective diagnostic tool to predict trapped lung, thereby helping to guide clinical decision-making regarding pleural interventions.

In this study, TUS was performed on patients scheduled for thoracentesis to assess the presence of the sinusoid sign, a pattern observed in the movement of the lung within the pleural fluid. Pleural pressures were simultaneously recorded using a digital manometer during thoracentesis at pre-determined volume intervals of fluid drainage. Trapped lung was diagnosed based on specific pleural pressure criteria: an initial pressure of −10 cmH_2_O, a pressure drops greater than 20 cmH_2_O per 1000 mL of fluid removed, pleural pressure below −25 cmH_2_O, or a PEL greater than 15 cmH_2_O. The peak and trough of the sinusoid wave observed on US were measured (delta), and this value was compared to the pleural manometry findings to assess the correlation between the two.

A total of ten patients were included in the study. Four cases exhibited a pleural pressure pattern consistent with trapped lung and were associated with an absent sinusoid sign (mean delta 0.08 cm). This was in contrast to four other cases in which minimal changes in pleural pressure, indicative of a mobile and expanding lung, were observed, and these cases were associated with a clear sinusoid sign (mean delta 0.98 cm). Additionally, two cases demonstrated pleural manometry findings suggestive of lung entrapment, but these cases had a less pronounced sinusoid sign (mean delta 0.53 cm).

The study concluded that the sinusoid sign, as assessed by TUS, shows potential for predicting underlying pleural physiology, specifically distinguishing between expandable and non-expandable lung. The data suggest that the presence or absence of the sinusoid sign correlates with pleural pressure patterns indicative of trapped lung or mobile lung, respectively. The authors emphasized that the ability to diagnose trapped lung using ultrasound could reduce the need for unnecessary bedside procedures, thereby minimizing associated complications.

Leemans et al. [17] conducted a retrospective study to investigate clinical and thoracoscopic predictors of successful talc pleurodesis in patients with MPE, aiming to guide patient selection and improve treatment outcomes. The study analyzed 155 symptomatic patients who underwent talc pleurodesis via medical thoracoscopy between January 2012 and December 2015. Of these, 122 patients (78%) achieved successful pleurodesis based on clinical and radiological criteria.

Univariate analysis identified several factors associated with unsuccessful pleurodesis. The presence of pleural adhesions (odds ratio (OR): 0.43, 95% CI: 0.19–0.96, *p* = 0.04), extensive spread of pleural lesions (OR: 0.17, 95% CI: 0.05–0.59, *p* = 0.001), use of systemic corticosteroids (OR: 0.28, 95% CI: 0.10–0.83, *p* = 0.02), and a prolonged delay between the clinical diagnosis of PE and the pleurodesis procedure (OR: 0.14, 95% CI: 0.06–0.32, *p* < 0.0001) were all significantly associated with pleurodesis failure. Among these, delayed pleurodesis emerged as the most important independent predictor of failure in multivariate analysis (OR: 0.08, 95% CI: 0.01–0.25, *p* < 0.0001).

TUS performed prior to pleurodesis was highlighted as a valuable tool for predicting procedural success. Between January and December 2015, TUS was systematically utilized in 30 patients to evaluate lung expandability. Using M-mode imaging, the atelectatic lung at the posterior chest base was visualized in the sitting position during breath-hold at functional residual capacity. Cine loops were acquired over at least three cardiac cycles, and the top-to-trough distance of the undulating pleural line was measured in millimeters. A cutoff value of <2 mm for this measurement was established based on receiver-operating characteristic analysis, providing a sensitivity of 91% and a specificity of 88% in predicting incomplete lung expansion after pleurodesis.

The study demonstrated a strong correlation between M-mode measurements and pleurodesis success (r = 0.69, 95% CI: 0.44–0.84, *p* < 0.0001), highlighting the utility of this method in assessing lung expandability and detecting pleural adhesions. These findings corroborate previous research by Salamonsen et al. [15], who emphasized the role of thoracic ultrasound in identifying trapped lung through the analysis of tissue movement and deformation.

Despite its strengths, including the use of TUS as an objective assessment tool, the study’s retrospective design and limited validation cohort were notable limitations.

Wong et al. [18] reported a single case report of a 50-year-old woman with metastatic breast carcinoma who developed recurrent right PE. Pre-procedure TUS showed an absent sinusoid sign, later confirmed by pleural manometry as indicative of trapped lung. The authors suggested that this non-invasive finding could serve as an early diagnostic marker, potentially reducing the need for pleural manometry. However, as this remains an isolated case, its broader applicability is uncertain and requires validation in further studies.

D.D. Herman et al. [19] responded to Wong et al.’s article with a letter to the editor, offering a broad evaluation that analyzed both the single case reported by Wong et al. and previous studies. While they acknowledged the value of a non-invasive method for predicting trapped lung and reducing the risks associated with pleural manometry and thoracentesis, they also identified significant limitations in the existing evidence.

Firstly, they questioned the reliability of the absent sinusoid sign due to its reliance on a non-peer-reviewed American Thoracic Society (ATS) conference abstract as a primary source (such as Wong’s case report). The lack of peer-reviewed validation raised concerns about applying this marker in clinical practice.

Beyond their critique of Wong et al.’s case, Herman et al. also evaluated the study by Flora et al., highlighting major methodological issues. They noted that the small sample size of only 10 patients, with only four cases used to derive the mean delta values for the sinusoid sign, limits the reliability of the conclusions. Additionally, the absence of variability measures (e.g., standard deviation) further weakened the robustness of the findings. The small absolute difference in delta values (0.08 cm for trapped lung vs. 0.98 cm for expandable lung) was particularly problematic, especially given that the intermediate value for entrapped lung (0.53 cm) was derived from only two patients, lacking variability data.

Another major concern was the lack of prospective validation of ultrasound measurements against the gold standard—pleural manometry. Without this validation, key diagnostic metrics such as sensitivity and specificity could not be determined, limiting the clinical applicability of the absent sinusoid sign. Herman et al. also suggested that effusion size could act as a confounding factor, as larger effusions (such as in trapped lung) might mechanically restrict pleural motion, potentially affecting the diagnostic performance of the sign.

In conclusion, while the absent sinusoid sign remains an interesting potential diagnostic tool, Herman et al. cautioned against its immediate clinical adoption. They emphasized the need for further peer-reviewed studies to validate its diagnostic value and address the methodological limitations before integrating it into routine clinical practice.

In their reply to D.D. Herman et al., Hassan Patail, Ada Wong, and Sahar Ahmad [20] acknowledged the constructive feedback and concerns raised regarding their case discussion of the absent sinusoid sign. They emphasized that while they appreciated the thoughtful review, the significance of ultrasound findings like the sinusoid sign, and its absence, warrants ongoing discussion and investigation.

The authors clarified that their study was not meant to draw definitive conclusions about the absent sinusoid sign based on a single case but rather to highlight the potential role of bedside TUS in assessing pleural disease. They emphasized that pleural manometry remains the gold standard for diagnosing trapped lung.

The authors stressed that the key takeaway from their article should be the expanding potential of bedside TUS for pleural disease diagnosis and management. They urged caution in interpreting case-based publications, underscored the need for validation, and expressed gratitude for the interest in their work, reaffirming their commitment to studying the absent sinusoid sign in pleural disease management.

Hassan et al. [21] conducted a study to investigate TUS predictors of NEL following pleural effusion drainage, focusing particularly on M-mode displacement (D) of the collapsed lung and lung/liver echogenicity (LLE) as potential markers. The study aimed to validate the utility of these predictors in patients undergoing medical thoracoscopy (MT), where complete drainage of the PE permits a reliable assessment of lung re-expansion.

In this study, 29 patients (59% female, mean age 49.6 years) with PE were examined pre-procedure using TUS at the posterior axillary line while seated. During breath-hold, M-mode was utilized to quantify the displacement of the collapsed lower lobe over several cardiac cycles. NEL was assessed post-procedure using CXR.

Of the 29 patients, NEL was identified in 8 patients (28%) after effusion drainage. Analysis of M-mode displacement as a predictor of NEL yielded an AUC of 0.48 (95% CI: 0.25–0.71, *p* = 0.86), indicating poor predictive value. A displacement cutoff of <1.7 mm showed a sensitivity of 62% and specificity of 54% in identifying NEL. In contrast, the LLE ratio demonstrated a stronger predictive performance, with an AUC of 0.77 (95% CI: 0.55–1, *p* = 0.03).

The study concluded that the previously reported ability of M-mode displacement to predict NEL may have been overstated, likely due to reliance on data from procedures where complete effusion drainage was not performed. Conversely, the LLE ratio emerged as a potentially valuable predictor of NEL, reflecting the stiffness of a poorly de-aerated lung, though further validation is required to confirm its clinical utility. This study highlights the limitations of M-mode displacement alone in predicting lung re-expansion and suggests the need for a more comprehensive approach to pre-procedural assessment of NEL.

Khatim et al. [22] presented a case of a 65-year-old male with small cell lung cancer and recurrent MPE, experiencing worsening dyspnea. CT revealed a large PE, while bedside TUS showed a significant anechoic space in the left thorax. M-mode imaging during breath-hold demonstrated reduced lung displacement due to cardiac impulses, indicating blunted cardiophasic variability suggestive of NEL.

The patient underwent ultrasound-guided placement of an IPC with 1200 mL of fluid drained. A post-procedure CXR showed pneumothorax ex vacuo, which resolved spontaneously without intervention. Follow-up imaging confirmed lung volume loss, further supporting the diagnosis of NEL.

The report emphasized the importance of pre-procedural identification of NEL to avoid ineffective pleurodesis and minimize complications, such as drainage-associated pneumothorax. M-mode TUS, which can detect blunted cardiophasic variability, was highlighted as a reliable, non-invasive alternative to traditional methods. The authors concluded that ultrasound provides a safer and efficient tool for diagnosing NEL, aiding in optimal management of patients with MPE.

Petersen et al. [23] conducted a prospective observational study to investigate the utility of TUS, specifically M-mode, in diagnosing NEL prior to thoracentesis in patients with suspected or known MPE. The study aimed to determine if TUS, particularly through the measurement of lung movement during respiration, could predict NEL, aiding clinicians in the management and treatment planning of affected patients.

The study involved 49 patients, who underwent ultrasound assessments at the site of planned thoracentesis. These assessments included B-mode, M-mode, and 2D shear wave elastography (SWE) measurements. M-mode, which measures lung movement through a real-time, dynamic assessment of the visceral pleura, provided the most promising results, with an AUC of 0.81, indicating strong diagnostic potential. In comparison, SWE and B-mode demonstrated lower diagnostic accuracy for detecting NEL.

M-mode ultrasound, by measuring the amplitude of lung movement, allowed for effective differentiation between expandable and non-expandable lung conditions. This technique showed high sensitivity and specificity, with clinical cutoffs that could guide treatment decisions, such as the use of IPCs for symptom relief in NEL patients. The results of the study suggest that M-mode, a relatively simple and widely available ultrasound modality, should be considered a core skill for respiratory physicians, particularly those managing pleural diseases.

The authors concluded that M-mode outperforms B-mode and SWE in identifying NEL before thoracentesis, offering a reliable, non-invasive diagnostic tool that aids procedural decisions and prevents unnecessary interventions. They acknowledged limitations, including a small sample size (49 patients) requiring validation in a larger, more diverse cohort. They also noted that the single-center setting introduced variability in operator experience and equipment, affecting reproducibility. To establish M-mode as a standard diagnostic tool, they emphasized the need for multicenter studies with larger populations to refine clinical cutoff values and enhance its accuracy in pleural disease management.

A summary table illustrating studies on the M-mode sinusoid sign assessment in TUS pre-procedural NEL assessment is provided below (Table 2).

### 5.2. Additional Emerging Preliminary Experimental Methods

#### 5.2.1. 2D Shear Wave Elastography (SWE)

Ultrasonographic elastography, first introduced in the 1990s, has become a standard tool in the evaluation of liver fibrosis and breast lesions [47]. Among its applications, ultrasound 2D shear wave elastography (SWE) enables the assessment of tissue stiffness based on acoustic resilience. Pathological tissues exhibit distinct mechanical properties compared to surrounding healthy tissue, with stiffer tissues allowing faster shear wave propagation [48]. SWE has demonstrated utility in distinguishing benign from malignant breast lesions [49], evaluating subpleural solid masses [50], and assessing hilar lymph nodes [51]. However, current clinical guidelines do not include SWE in the diagnostic work-up for suspected NEL [52,53].

Petersen et al. [23] hypothesized that SWE might outperform conventional B-mode and M-mode ultrasonography in diagnosing NEL, particularly in patients with suspected MPE, by identifying differences in tissue elasticity between normal and fibrotic pleural lining. To test this hypothesis, measurements were obtained from three structures: the parietal pleura, pleural effusion above the lung, and the visceral pleura or consolidated lung tissue. Each structure was measured five times, with minimal transducer pressure applied to the chest wall to avoid distortion. Measurements were recorded during breath-hold, with a region of interest (ROI) carefully selected on the elastogram to ensure optimal shear wave propagation. ROIs were adjusted in size as necessary, and results were expressed in meters per second (m/s) [54].

Despite these efforts, SWE was found to be less effective than M-mode measurements of lung movement and B-mode assessment of diaphragm movement in predicting NEL, with AUC values of 0.81, 0.77, and 0.65, respectively. The AUCs for SWE measurements were notably lower: 0.57 for the parietal pleura, 0.47 for the pleural effusion, and 0.59 for the visceral pleura.

This study represents the first evaluation of 2D SWE for diagnosing NEL. The findings indicate that SWE is less effective than conventional M-mode and B-mode TUS in assessing lung and diaphragm movement as predictors of NEL. These results highlight the current limitations of SWE in this context and suggest that, for now, traditional ultrasound techniques remain more reliable for clinical practice. However, further studies on elastography techniques are necessary to fully explore their potential utility in NEL evaluation. Additional research is highly warranted to better define the role of SWE in this clinical setting.

#### 5.2.2. Speckle Tracking Imaging (STI) Strain Analysis

Speckle tracking imaging (STI) is an advanced echocardiographic technique that analyzes myocardial deformation by tracking the movement of speckles within the ultrasound images [55]. Originally developed to assess myocardial contractility, STI has gained recognition for its ability to provide quantitative data on strain and motion, offering insights into cardiac function [56]. The technique utilizes two-dimensional echocardiography to measure the displacement of ultrasound speckles over time, allowing for the calculation of strain in various cardiac structures. As a result, STI has become an essential tool in cardiology, aiding in the diagnosis and management of various cardiac conditions [55,56].

Literature highlights the expanding applications of STI beyond cardiac assessment, also in evaluating lung conditions, such as NEL. Salamonsen et al. [15] employed STI using TUS to assess the movement and strain of the atelectatic lung prior to PE drainage. The procedure involved obtaining targeted TUS images of the ipsilateral lower lobe, followed by strain analysis through the EchoPAC software. The strain data were acquired during breath-holding at functional residual capacity, and the analysis was performed by an experienced cardiologist blinded to clinical and radiologic information. Results demonstrated that STI provided a high diagnostic accuracy with an AUC of 0.86 for detecting entrapped lung, outperforming other methods such as M-mode ultrasound (AUC = 0.79) and PEL (AUC = 0.69). STI showed strong sensitivity (71%) and specificity (85%) for detecting entrapped lung, and the study highlighted that the amount of strain observed was significantly reduced in entrapped lung due to the lack of lung movement. However, factors such as lung location relative to the heart and partial atelectasis may influence STI readings and potentially lead to false positives. Despite these limitations, STI’s ability to non-invasively diagnose entrapped lung holds promise for guiding clinical decision-making. The study’s findings suggest that STI could become an integral tool in the management of MPE, although further research is required to refine the technique and overcome current limitations, including the impact of out-of-plane motion in two-dimensional imaging.

#### 5.2.3. Lung/Liver Echogenicity (LLE) Ratio

Echogenicity, the ability of tissues to reflect ultrasound waves, can be quantitatively assessed using image processing tools such as ImageJ (1.54m) (NIH) [57]. This software facilitates the detailed analysis of ultrasound images, enabling the measurement and comparison of echogenicity levels across different tissues. Widely applied in medical fields, ImageJ is particularly valuable in evaluating muscle quality, kidney conditions, and other soft tissue characteristics. Clinically, it is used to calculate echogenicity ratios between organs, providing insights into tissue composition, pathology, and functional status [58,59,60].

The process of calculating echogenicity involves several steps: first, acquiring the ultrasound image; second, selecting the ROI by identifying and tracing the target area on the image, such as the PE region; and third, using ImageJ to quantify pixel density within the ROI on a 0–100 grayscale scale, which reflects the echogenicity of the analyzed tissue.

Hassan et al. [21] investigated ultrasound-based predictors for lung re-expansion after PE drainage, focusing on lung/liver echogenicity (LLE) ratio as a novel parameter. Building on prior work on M-mode ultrasound for assessing collapsed lung displacement during breath-hold as a predictor of NEL, the study addressed the limitations of incomplete effusion drainage and evaluated LLE’s predictive value after complete drainage via medical thoracoscopy (MT). LLE reflects lung stiffness and aeration status, as healthy, aerated lung tissue has low echogenicity due to air scattering, while diseased, non-aerated lung tissue exhibits higher echogenicity, resembling solid organs like the liver.

**Figure 5:**
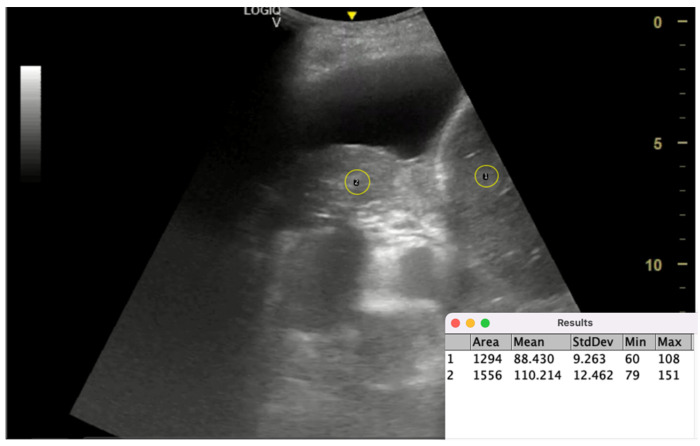
The ratio between the echogenicity of a collapsed lung and the liver (LLE) was quantified using TUS images with the assistance of software called ImageJ. In this example, the LLE is calculated as 110.214/88.439 = 1.24. M. Hassan et al. propose a cutoff value of 1.65 or higher to predict non-expandable lung (NEL), with a sensitivity of 83% and specificity of 77.5%.

In this study, 29 patients with PE undergoing MT with complete effusion drainage were assessed. Pre-procedure, TUS was performed with patients seated at the posterior axillary line. During breath-hold, TUS M-mode measured the displacement (D) of the collapsed lower lobe over several cardiac cycles, while the echogenicity of the collapsed lung and liver was quantified using ImageJ software (Figure 5). The LLE ratio was hypothesized to be a marker of lung stiffness, indicating the failure of lung de-aeration despite effusion drainage and potentially predicting NEL.

Post-procedure CXRs identified NEL in 8 patients. Results showed M-mode displacement had poor predictive value (AUC = 0.48), while LLE demonstrated superior performance (AUC = 0.77). An LLE cutoff > 1.6 yielded 71% sensitivity and 83% specificity for predicting NEL. These findings suggest LLE effectively reflects lung de-aeration and stiffness, outperforming M-mode displacement in predicting NEL when effusion drainage is complete.

Despite its promise, the study’s small sample size and reliance on CXRs as the standard for lung re-expansion assessment limit its generalizability. Larger, multicenter studies are needed to validate LLE as a routine predictive tool. Nevertheless, this research highlights LLE’s potential to enhance non-invasive lung stiffness assessment, guide therapeutic decisions, and improve outcomes for patients with PE.

#### 5.2.4. The Dynamic Air Bronchogram

The dynamic air bronchogram is a well-established ultrasound sign useful in diagnosing pulmonary conditions such as pneumonia and atelectasis. First described by Dr. Lichtenstein [61], it appears as hyperechoic air-filled bronchi moving synchronously with respiration within consolidated lung tissue. This movement indicates airway patency, distinguishing it from static air bronchograms, which suggest obstruction [62].

Dynamic air bronchograms are particularly valuable in critical care settings where conventional imaging is limited. They provide a non-invasive, bedside diagnostic tool, avoiding radiation exposure from CT scans and logistical challenges of patient transfer [62,63]. Their presence strongly suggests pneumonia over atelectasis, with studies reporting high specificity (94%) and positive predictive values (97%) [61].

Recent literature highlights their utility in various clinical contexts, including ventilator-associated pneumonia, where studies have demonstrated 100% specificity [64].

**Figure 6:**
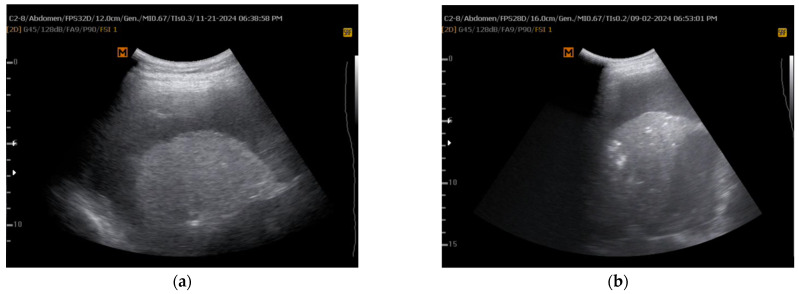
TUS images of: (**a**) lung atelectasis without a dynamic air bronchogram; (**b**) dynamic air bronchogram. TUS images courtesy of Hüseyin Yildirim [24].

Yıldırım et al. [24] investigated the diagnostic role of dynamic air bronchograms in detecting NEL. The study included 45 patients (mean age 65.4 years), 28 with malignant and 17 with benign PE. TUS, performed in a sitting position, assessed dynamic air bronchograms—visualized as echogenic columns within atelectatic lung (Figure 6, courtesy of Hüseyin Yildirim)—before draining 1000 mL of pleural fluid. NEL was confirmed via clinical observation and post-thoracentesis CXRs at 4 and 24 h. Among participants, 18 (40%) were diagnosed with NEL. Dynamic air bronchograms demonstrated high diagnostic performance, with sensitivity of 92.5%, specificity of 83.3%, accuracy of 88.8%, positive predictive value of 89.2%, and negative predictive value of 88.2%. These findings suggest that dynamic air bronchograms can serve as a simple, effective, and non-invasive marker for NEL, potentially complementing other ultrasound assessments and diagnostic methods. However, as these are only preliminary data, further high-quality studies are needed for robust validation.

#### 5.2.5. Pleural Thickening

Regarding effusion type on TUS, Faber and Krenke (2021) found a significant link between complex septated effusions and entrapped lung (*p* < 0.05) [35].

Pleural thickening > 0.5 cm on TUS showed a statistically significant difference between the entrapped and non-entrapped lung groups [65]. In the entrapped lung group, all patients (100%) had pleural thickening > 0.5 cm, whereas in the non-entrapped lung group, 50% exhibited pleural thickening and 50% had none (*p* = 0.0025).

These findings are consistent with previous research. Huggins et al. (2007) reported significantly abnormal visceral pleural thickening on high-resolution CT in entrapped lung cases [25]. Similarly, Light et al. (1980) demonstrated a strong association between pleural thickening and trapped lung, a subset of the entrapped lung group [45].

A summary table illustrating additional emerging preliminary experimental methods for pre-procedural NEL assessment is provided below (Table 3).

## 6. Management of Non-Expandable Lung in Malignant Pleural Effusion: Treatment Strategies and Patient-Centered Care

### 6.1. Treatment Strategies for Non-Expandable Lung

The management of symptomatic MPE in patients with NEL presents significant challenges, affecting approximately 30% of cases. Expert consensus defines NEL as radiologically significant when >25% of the lung remains unopposed to the chest wall on CXR after pleural drainage [4]. Interobserver variability in radiograph interpretation exists, and NEL is associated with a worse prognosis in MPE [66].

The American Thoracic Society (ATS) recommends IPCs as the treatment of choice for MPE in the setting of NEL, emphasizing that chemical pleurodesis, particularly talc pleurodesis, is rarely effective in these cases [67]. For patients with a predicted short survival, ATS advises a palliative care approach focusing on symptom management, including repeat thoracentesis, oxygen therapy, and morphine for breathlessness [67]. Similar recommendations are made by the European Respiratory Society (ERS), European Association for Cardio-Thoracic Surgery (EACTS), and the Spanish Society of Pulmonology and Thoracic Surgery (SECT), all advocating for IPCs in cases involving NEL [52,68].

In contrast, the 2023 guidelines from the British Thoracic Society (BTS) propose a stratified approach based on the degree of lung re-expansion. For patients with more than 25% of the lung not expanding, IPCs are preferred over talc pleurodesis, while those with less than 25% non-expandable lung may benefit from either IPC or talc slurry pleurodesis, based on clinical presentation and patient preference [4]. While surgical interventions are not routinely recommended, decortication may be considered in highly selected patients with trapped lung where lung expansion is a critical goal. BTS also acknowledges thoracentesis as a possible option but recommends discussing alternatives due to the potential need for repeated procedures [4].

The clinical question addressed is whether pleural aspiration, talc slurry pleurodesis, talc poudrage pleurodesis, or decortication surgery is superior to IPC in improving clinical outcomes in adults with MPE and NEL.

Outcome measures for evaluating treatment efficacy include QoL, length of hospital stay, need for re-intervention, symptom relief (such as breathlessness and chest pain), complications, and pleurodesis success rates. Current evidence, however, does not directly compare the different treatment modalities for MPE with NEL, and as such, no definitive recommendations can be made.

The evidence review suggests that while IPCs may improve QoL and alleviate breathlessness, they often remain in situ for prolonged periods, with some studies reporting an average catheter duration of 94 days [69]. Although the risk of pleural infection is small, it remains a consideration, with a 4.9% infection rate reported, mostly manageable with antibiotics [70]. Spontaneous pleurodesis has been observed in 48% of patients with IPCs [69]. Talc slurry pleurodesis and talc poudrage pleurodesis have not been directly compared to IPC in the context of NEL, and the limited available studies do not provide sufficient evidence to favor one approach over the other. Decortication surgery, although not compared directly to IPC in randomized controlled trials, has been associated with improved pleurodesis success rates in some retrospective studies [71].

In conclusion, treatment decisions should be individualized based on patient preferences, clinical condition, and available evidence, with careful consideration of the risks and benefits associated with each approach.

### 6.2. Patient Education, Shared Decision-Making, and Coordinated Care

Educating patients about NEL is crucial to preventing unnecessary interventions and optimizing outcomes. A multidisciplinary approach involving pulmonologists, thoracic surgeons, and radiologists ensures comprehensive care. Active patient engagement in decision-making helps select the most appropriate treatment [72]. Providing clear, accessible information—whether verbal, written, or digital—empowers patients to make informed choices based on their preferences, goals, and risk–benefit considerations. Ongoing discussions with patients and caregivers address concerns early, maximizing therapeutic benefits and improving outcomes.

Given institutional variability in clinical practice, standardized guidelines should serve as a foundation for treatment decisions, ensuring that patient-specific factors guide management rather than institutional preferences [4]. Patients must be fully informed about the risks and benefits of each treatment option, enabling evidence-based decision-making that aligns with the best possible care.

A comprehensive, individualized approach to NEL management is essential to optimize patient outcomes, minimize complications, and reduce healthcare costs. Both IPCs and talc pleurodesis require vigilant monitoring and proactive management of complications to ensure optimal therapeutic efficacy [73]. This careful attention to treatment plans and early intervention in response to complications enhances patient well-being and minimizes the financial burden on healthcare systems.

## 7. Medical Consequences, Complications, and Healthcare Costs of Inadequate NEL Management

### 7.1. Impact on Quality of Life and Clinical Outcomes

Inadequate management of NEL can have profound consequences on both patient well-being and healthcare efficiency. Persistent symptoms such as breathlessness and chest discomfort can significantly limit daily activities, mobility, and overall QoL [5]. These symptoms, along with unresolved PEs, may contribute to anxiety and further reduce the patient’s functional capacity, leading to a diminished sense of well-being.

The inability to effectively manage NEL often results in prolonged hospital stays. Recurrent pleural procedures, infections, or complications from interventions contribute to extended hospitalizations, escalating both the financial and healthcare burden. Additionally, unresolved effusions, failed pleurodesis, or recurring symptoms may necessitate repeated thoracenteses, IPCs, or even surgical interventions, each of which exposes the patient to additional risks and increases the complexity of care [74].

Poorly controlled NEL leads to chronic dyspnea and chest discomfort, which severely impair patient comfort and limit physical activity. The progression of these symptoms can result in further deterioration of respiratory function, ultimately contributing to progressive respiratory failure. Failure to adequately control symptoms, particularly in the absence of effective interventions, exacerbates these challenges, affecting both patient comfort and long-term outcomes.

Pleurodesis failure is a common complication in patients with NEL, with ineffective or inappropriate attempts leading to high failure rates. In such cases, alternative interventions like IPCs or surgical procedures such as decortication may be required to manage the condition [75,76,77]. These alternatives, while sometimes necessary, bring added complexity and risk, reinforcing the importance of an individualized and tailored approach to patient management.

### 7.2. Procedural Complications and Adverse Events

Both IPCs and talc pleurodesis are associated with distinct adverse event profiles, though a meta-analysis found no significant overall difference in complication rates between the two modalities [78]. IPCs carry an increased risk of infection due to prolonged foreign body presence, with skin or pleural fluid infections occurring in approximately 8% of cases [79]. While IPC-related infections are notable, they generally resolve with appropriate antibiotic therapy without necessitating catheter removal [67,68]. In cases of worsening infection, escalation to intravenous antibiotics, hospitalization, or catheter removal may be required [67]. Interestingly, some pleural infections lead to spontaneous pleurodesis (autopleurodesis). Other IPC-related complications include catheter obstruction (≈5%) and the formation of pleural septations (<15%) [68,79].

Adverse events linked to talc pleurodesis include pain, dyspnea, infection, post-procedural fever, tube obstruction, and displacement, occurring in fewer than 10% of cases [76,77,80]. The risk of acute respiratory distress syndrome, historically associated with small talc particles, has been mitigated using graded talc [81].

Both IPCs and talc pleurodesis require vigilant monitoring and proactive management of complications to optimize patient outcomes.

### 7.3. Cost-Effectiveness Considerations

The economic impact of NEL management varies based on regional healthcare policies and resource availability. While in well-funded healthcare systems, such as those in Europe and the United States, treatment selection prioritizes patient suitability and clinical factors, in lower-income settings, cost-effectiveness plays a more substantial role in decision-making [82,83]. Both talc pleurodesis and IPCs reduce the need for repeated pleural interventions compared with thoracentesis, making them more cost-effective in patients without a very limited life expectancy [84]. IPCs, in particular, are associated with reduced inpatient stays and fewer subsequent interventions compared to talc pleurodesis [76,77], generating significant cost savings. However, in overcrowded healthcare systems with limited hospital bed availability, logistical constraints may restrict access to optimal treatment, potentially denying some patients the most appropriate therapeutic option.

## 8. Discussion

The clinical significance of NEL in the context of MPE is critical, as it directly influences patient management and prognosis. NEL, resulting from trapped lung or lung entrapment, complicates lung re-expansion and often leads to suboptimal responses to standard treatments like pleurodesis.

Diagnosing NEL remains challenging due to the lack of standardized criteria. Clinical symptoms such as excessive coughing, progressive dyspnea, chest pain, and general discomfort from reduced intrathoracic pressure may suggest NEL during thoracentesis [1,29,30,31,32,33]. Radiological signs, including visceral pleural thickening and (hydro)-pneumothorax, are more easily identifiable in post-thoracentesis imaging, often serving as primary diagnostic indicators. However, these signs are detected only after thoracentesis, risking delayed or missed diagnosis [41,42].

Several studies have suggested pleural manometry as a potential diagnostic standard for NEL [8,25,36,37,38]. However, standardization of methods and threshold values across studies is needed, and current guidelines do not recommend pleural manometry as a reference standard nor demonstrate its ability to prevent pleural pressure-related complications during thoracentesis [9,35]. Diagnosis is therefore often established during the peri- and post-procedural stages, leading to suboptimal NEL management, exacerbating medical complications, and increasing healthcare costs.

Prior awareness of NEL could guide procedural approaches and therapeutic decisions in patients with PEs, highlighting the urgent need for further research to develop early, pre-procedural, non-invasive diagnostic methods for NEL. Recent studies highlight significant advancements in the non-invasive pre-procedural assessment of NEL in MPE, revealing promising diagnostic modalities that may refine clinical decision-making.

Non-invasive, real-time, bedside modalities such as M-mode TUS during breath-hold have shown efficacy in predicting NEL by detecting an absent sinusoidal sign and reduced atelectatic lung movement [13,14,15,16,17,18,19,20,21,22,23]. The integration of bedside TUS into clinical practice could reduce unnecessary interventions like repeated thoracentesis or ineffective pleurodesis, which can cause pneumothorax and improve patient outcomes by personalizing treatment based on the likelihood of successful lung re-expansion.

However, limitations exist, including small sample sizes, single-center settings, and variability in operator expertise and equipment, warranting caution in interpretation [10,11,12]. While M-mode ultrasound has shown promise, concerns remain regarding its accuracy, with some studies suggesting overstated predictive ability. The absent sinusoid sign requires further validation before clinical use. Additional peer-reviewed studies are necessary to refine its diagnostic capabilities.

Future research should focus on refining diagnostic criteria and clinical cutoff values for M-mode ultrasound and the sinusoid sign. Large-scale, multicenter randomized trials are essential to clarify the role of these tools in predicting NEL and improving decision-making. Exploring the combined use of ultrasound with pleural manometry or biomarker analysis may provide more accurate predictions. Standardized protocols for TUS interpretation and training programs for operator consistency will maximize clinical utility.

Given these limitations, there is a clear and pressing need for further research to develop advanced, non-invasive strategies for the early diagnosis of NEL. In light of this, recent years have seen the emergence of additional experimental imaging techniques aimed at optimizing NEL prediction at the earliest possible stage [15,21,23,24,25].

Speckle tracking imaging (STI) strain analysis has demonstrated high diagnostic accuracy (AUC = 0.86), outperforming M-mode ultrasound and pleural elastance in detecting entrapped lung [15]. This quantitative, non-invasive technique offers advantages over traditional ultrasound markers. However, its clinical utility is limited by several factors, including dependency on lung location, susceptibility to motion artifacts and out-of-plane movement, and the requirement for specialized software analysis. Additionally, its availability is largely restricted to cardiology settings. Despite these limitations, STI strain analysis holds significant potential for NEL evaluation and warrants further investigation [15].

The lung/liver echogenicity (LLE) ratio, a quantitative imaging technique using ImageJ software, has shown promise in predicting lung re-expansion after pleural drainage (LLE > 1.6, AUC = 0.77) [21]. While simple and reproducible, its clinical applicability is limited by small study populations, image quality dependence, and the need for grayscale calibration. As it requires specialized software and moderate operator expertise, further validation through larger, multicenter studies, potentially integrating AI-based analysis, is warranted [21].

Two-dimensional shear wave elastography (SWE) has proven less effective than M-mode and B-mode ultrasound in assessing lung and diaphragm movement as predictors of NEL [23]. Although SWE has demonstrated limited performance, its potential warrants further investigation. Additional studies are required to fully explore the utility of elastography techniques and better define their role in NEL assessment [23].

Assessed via TUS, the dynamic air bronchogram, which visualizes air-filled bronchi in consolidated lung tissue, has demonstrated 92.5% sensitivity and 83.3% specificity for NEL [24]. It is bedside-performed and non-invasive. However, it may result in false positives and is limited by poor lung ultrasound windows. Given these preliminary findings, further high-quality studies are needed for robust validation [24].

TUS evaluation of pleural thickening > 0.5 cm is strongly associated with entrapped lung but lacks specificity due to the influence of chronic inflammation [25]. It is less effective than CT in assessing visceral pleural thickening. While easy to perform with a high-frequency probe and dependent on operator skill, it remains a promising technique for further investigation and potential integration with other TUS signs [25].

As previously mentioned, it is clear that each of these emerging pre-procedural techniques requires high-quality validation before they can potentially be routinely integrated into clinical practice for the diagnosis of NEL. However, if further refined and validated through rigorous research, these methods hold the potential to significantly enhance clinical decision-making. Given the clinical need, it is essential to develop new studies focused on these techniques, alongside evaluating advanced imaging technologies, to explore their potential in the early diagnosis and management of NEL.

A multimodal diagnostic strategy integrating clinical manifestations, pleural manometry, radiologic and ultrasonographic findings, and emerging techniques (once fully validated) may offer the most robust and comprehensive framework for NEL.

In this understudied area, AI holds the potential to revolutionize NEL management by addressing challenges in both diagnosis and treatment. While underrepresented in NEL research, AI could process multimodal datasets from imaging techniques like TUS, CXRs, and CT scans, identifying patterns in lung mechanics and pathology that may go undetected [85,86,87]. Integrating AI with real-time diagnostic systems could forecast lung re-expansion likelihood, aiding clinicians in making data-driven decisions [88]. Future investigations should refine AI models using diagnostic data, including clinical presentation, imaging, ultrasound results, pleural manometry, and biomarkers. These predictive models could offer individualized assessments of re-expansion potential, optimizing treatment selection and improving outcomes. Identifying novel biomarkers specific to NEL and incorporating them into AI models could enhance diagnostic precision and inform targeted therapies.

Standardizing TUS protocols and developing training programs to ensure consistent interpretation of ultrasound in NEL diagnosis will be crucial for widespread adoption. Exploring non-invasive monitoring methods, like novel imaging technologies [89] and wearable devices [90,91], could offer real-time insights into disease progression, reducing the need for repeated procedures.

Cost-effectiveness assessments of emerging diagnostic technologies and therapeutic interventions are essential to optimize healthcare resource allocation, particularly in resource-constrained settings. These evaluations enable the identification of the most effective and economically viable approaches, ensuring that novel technologies are incorporated into clinical practice in a manner that maximizes their clinical benefit while minimizing unnecessary expenditures. By adopting such an approach, the sustainable integration of advanced diagnostic and therapeutic strategies across various healthcare systems can be supported, facilitating equitable access to high-quality care.

Regardless of potential diagnostic advancements in NEL, patient-centered care remains paramount. Key components of this approach include shared decision-making, tailored education on treatment adherence, and a focus on quality of life. It is crucial that patients are fully informed about the risks and benefits of treatment options, enabling them to make evidence-based decisions that align with their preferences and therapeutic goals. Ongoing discussions with patients and caregivers help identify concerns early, optimizing treatment outcomes and improving overall well-being. To ensure consistent and personalized care across healthcare institutions, standardized yet flexible guidelines are essential. These should be evidence-based and reproducible, while adaptable to the diverse conditions and preferences of individual patients. By combining these protocols with a patient-centered approach, treatments can be both effective and tailored to each patient’s needs, ultimately enhancing outcomes and overall care.

Continued research will be crucial to refine and validate emerging diagnostic techniques to establish reliable, non-invasive, pre-procedural methods for the early detection and personalized management of NEL. This effort will ultimately improve patient outcomes, reduce healthcare costs, and optimize treatment strategies in the long term.

## Figures and Tables

**Table 1:** Comparative analysis of imaging evaluation strategies in the pre-, peri-, and post-procedural assessment of non-expandable lung.

Aspect	Pre- and Peri-Procedural Evaluation	Post-Procedural Evaluation
Imaging modalities	- Upright and decubitus CXR for rapid and cost-effective assessment of NEL - Chest CT, providing high-resolution anatomical detail for early identification of pleural pathology - TUS for real-time, bedside dynamic assessment	- CXR (baseline follow-up) - TUS (detects complications - like pneumothorax) - CT, if further anatomical detail required
Key radiologic findings	- Evidence of pleural thickening and loculations even before pleural fluid removal - Pleural fluid quantification - Limited mobility of pleural fluid - Early signs indicative of a trapped lung - (i.e., reduced hemithorax size) - TUS dynamic assessment of lung expandability	- Basal pneumothorax (ex vacuo pneumothorax) frequently observed - Persistent pleural thickening - Incomplete lung re-expansion - despite pleural fluid drainage
Aims	- Pre-procedural imaging aids in procedural planning and predicting lung expandability	- Post-procedural imaging is for confirming the degree of lung re-expansion, and detects complications
Clinical impact and management	- Guides procedural planning by identifying features (e.g., pleural thickening, loculations) that suggest NEL - Predicts potential for lung re-expansion during thoracentesis	- Confirms the diagnosis of NEL by demonstrating persistent radiologic abnormalities after drainage - Helps monitor complications and guide subsequent therapeutic decisions
Limitations	- CXRs may miss subtle changes and two-dimensional view rays can obscure nuanced pleural details due to overlapping structures - Both CXR and CT lack dynamic evaluation (e.g., absence of lung sliding is not assessed), thus may underestimate the complexity of pleural adhesions - TUS is operator-dependent and provides a limited global anatomical perspective, necessitating complementary imaging techniques.	
Solutions	- A multimodal strategy incorporating clinical manifestations, pleural manometry, and various imaging techniques	

**Table 2:** Summary of studies on M-mode sinusoid sign assessment in TUS for pre-procedural NEL assessment.

Study	Year	Objective	Study Design	Population	Ultrasound Technique	Main Findings	Clinical Implications	Main Limitations
Lichtenstein et al. [13]	2008	Evaluate TUS in acute respiratory failure (BLUE protocol)	Prospective observational study	Critically ill patients with respiratory distress	M-mode, B-mode	Identified sinusoid sign as indicative of lung expansion	Standardized lung ultrasound use in emergency settings	Limited generalizability beyond emergency care
Salamonsen et al. [15]	2014	Assess M-mode US for predicting NEL	Prospective observational study	81 patients with MPE	M-mode, speckle tracking imaging (STI)	M-mode displacement <0.8 mm predictive of NEL	Allowed pre-drainage identification of entrapped lung	Some misclassification due to incomplete drainage
Flora et al. [16]	2017	Correlate sinusoid sign with pleural manometry	Prospective observational study	10 patients undergoing thoracentesis	M-mode	Presence of sinusoid sign correlated with lung re-expansion	Suggested US as non-invasive alternative to pleural manometry	Small sample size
Leemans et al. [17]	2018	Investigate predictors of successful talc pleurodesis in malignant pleurisy	Retrospective study	155 patients undergoing talc pleurodesis	M-mode	M-mode displacement <2 mm correlated with pleurodesis success (91% sensitivity, 88% specificity)	Highlighted role of M-mode in predicting pleurodesis success	Retrospective design, limited validation cohort
Wong et al. [18]	2019	Case report on absent sinusoid sign and trapped lung	Case report	1 patient with metastatic breast cancer	M-mode, B-mode	Absent sinusoid sign correlated with high pleural elastance	Suggested pre-thoracentesis US assessment	Single case, needs validation
Hassan et al. [21]	2021	Validate TUS predictors of NEL	Prospective cohort	29 patients with pleural effusion	M-mode, lung/liver echogenicity (LLE) ratio	M-mode alone was a poor predictor (AUC = 0.48), LLE ratio was better (AUC = 0.77)	LLE may serve as a better predictor for NEL than M-mode alone	Small sample size, single-center study
Khatim et al. [22]	2022	Diagnosis of NEL using TUS	Case report	1 patient with small cell lung cancer and recurrent MPE	M-mode	Blunted cardiophasic variability indicated NEL, confirmed by post-drainage imaging	Highlighted importance of pre-procedural TUS assessment for NEL	Single case, limited generalizability
Petersen et al. [23]	2024	Compare M-mode, B-mode, and SWE for NEL prediction	Prospective observational	49 patients with suspected MPE	M-mode, B-mode, 2D shear wave elastography (SWE)	M-mode had highest AUC (0.81) for NEL prediction	Reinforced M-mode as core tool for pleural disease assessment	Small sample, single-center variability

**Table 3:** Summary of additional emerging preliminary experimental methods for pre-procedural non-expandable lung assessment.

Method	Principle	Applications	Key Findings	Strengths	Limitations	Comparison to Gold-Standard Imaging	Standardization and Feasibility	Potential Future Directions
2D shear wave elastography (SWE)	Measures tissue stiffness by analyzing shear wave propagation	Assessing hilar lymph nodes, subpleural masses	Less effective than B-mode and M-mode in diagnosing NEL (AUC = 0.57–0.65)	Non-invasive, quantitative, widely used in other fields (e.g., liver, breast imaging)	Poor diagnostic performance for NEL, influenced by measurement variability	Inferior for lung tissue assessment; lacks histopathological confirmation	Requires specialized ultrasound equipment and training, ROI selection critical	Optimization of ROI selection, combination with other ultrasound markers, integration with AI algorithms
speckle tracking imaging (STI) strain analysis	Measures myocardial and lung tissue deformation using ultrasound speckle tracking	Assessing entrapped lung in MPE	High diagnostic accuracy (AUC = 0.86), superior to M-mode and pleural elastance	Quantitative, non-invasive, better than traditional ultrasound markers for entrapped lung	Affected by lung location, motion artifacts, out-of-plane movement	Superior to pleural elastance but lacks direct comparison to CT	Requires expertise in software analysis; not widely available outside cardiology settings	Standardization of acquisition protocol, use in combination with manometry and TUS
lung/liver echogenicity (LLE) ratio	Compares grayscale pixel density of lung and liver via ImageJ software	Predicting lung re-expansion after pleural drainage	LLE > 1.6 predicts NEL with AUC = 0.77	Simple, reproducible, quantitative	Limited validation, small study population, dependent on image quality	Lacks direct validation against; potential role as adjunct to manometry	Requires software (ImageJ) and proper grayscale calibration, moderately operator-dependent	Larger, multicenter studies, AI-based echogenicity analysis, validation in different effusion types
Dynamic air bronchogram	Visualization of air-filled bronchi moving in consolidated lung tissue	Differentiating pneumonia from atelectasis, predicting NEL	Sensitivity 92.5%, specificity 83.3% for NEL	High specificity, easily performed at bedside	Potential for false positives, limited by poor lung ultrasound window	Comparable to CT but more accessible; lacks histopathological confirmation	Widely feasible with standard ultrasound equipment, easily performed by trained operators	Further validation in different pleural disease contexts, integration with other TUS signs
Pleural thickening	Measurement of pleural thickness on TUS	Predicting entrapped lung and complex pleural effusions	Thickness >0.5 cm strongly associated with entrapped lung	Strong association with pathophysiology	Lacks specificity, influenced by chronic inflammation	Inferior to CT in evaluating visceral pleural thickening; lacks correlation with biopsy findings	Easy to perform, requires high-frequency probe, operator-dependent	Integration with pleural manometry, evaluation of histopathological correlation, potential role in guiding biopsy

## Data Availability

No new data were created or analyzed in this study. Data sharing is not applicable to this article.

## Abbreviations

The following abbreviations are used in this manuscript:

NEL	Non-expandable lung
PE	Pleural effusion
CXR	Chest X-ray
CT	Computed tomography
TUS	Thoracic ultrasound
M-mode	Motion mode
STI	Speckle tracking imaging
SWE	Shear wave elastography
MPE	Malignant pleural effusion
IPC	Indwelling pleural catheter
PEL	Pleural elastance
LLE	Lung/liver echogenicity ratio
MT	Medical thoracoscopy
AUC	Area under the curve
ROI	Region of interest
QoL	Quality of life
AI	Artificial intelligence
ATS	American Thoracic Society
ERS	European Respiratory Society
EACTS	European Association for Cardio-Thoracic Surgery
SECT	Spanish Society of Pulmonology and Thoracic Surgery
BTS	British Thoracic Society
